# Tissue Depletion of Olaquindox and Its Six Metabolites in Pigs and Broilers: Identification of a Suitable Marker Residue

**DOI:** 10.3389/fvets.2021.638358

**Published:** 2021-04-23

**Authors:** Heying Zhang, Wei Qu, Chaoyue Ding, Juncheng Han, Shuyu Xie, Zhenli Liu, Lingli Huang, Yuanhu Pan, Zonghui Yuan

**Affiliations:** ^1^National Reference Laboratory of Veterinary Drug Residues (HZAU), Huazhong Agricultural University, Wuhan, China; ^2^Ministry of Agriculture Laboratory of Risk Assessment for Quality and Safety of Livestock and Poultry Products, Huazhong Agricultural University, Wuhan, China

**Keywords:** olaquindox, metabolites, residue depletion, deoxyolaquindox, MQCA

## Abstract

The depletion profiles of olaquindox and its six major metabolites, including O1 (*N*^1^-deoxyolaquindox), O2 (deoxyolaquindox), O3 (2-carboxamide-3-methylquinoxaline-*N*^4^-oxide), O4 (2-carboxymethylaminocarbonyl-3-methylquinoxaline-*N*^4^-oxide), O5 (2-carboxymethylaminocarbonyl-3-methylquinoxaline), and O6 [3-methyl-quinoxaline-2-carboxylic acid (MQCA)] were studied with a sensitive and accurate HPLC-UV method in pigs and broilers after oral administration of olaquindox at the rate of 50 mg kg^−1^ feed for 14 consecutive days. Five medicated pigs and six medicated broilers and one control animal for each time point were anesthetized and killed at different time points (6 h and 1, 3, 7, and 14 days for pigs and 6 h and 1, 3, 5, and 7 days for broilers) after ingestion of the medicated feed ceased and samples of muscle, liver, kidney, and fat were collected. The samples were assayed using a liquid chromatographic method. Mean concentrations of O2 (deoxyolaquindox) metabolite residues in all tissues of pigs were higher than other metabolite residues at each time point. MQCA was detected at lower concentrations and eliminated more rapidly than deoxyolaquindox (calculated *t*_1/2_ 1.78–2.28 days vs. *t*_1/2_ 2.04–2.46 days). The elimination half-lives of deoxyolaquindox residue in broilers' liver and kidney tissues (*t*_1/2_ >4 days) were much longer than those in pigs. Thus, the use of olaquindox in poultry is clearly inappropriate, as significant drug residues will occur without a withdrawal time. The results that deoxyolaquindox occurs at higher concentrations in kidney tissue and is more persistent than other residues in edible tissues of pigs which indicate that deoxyolaquindox is the most relevant marker residue and should be monitored in the routine surveillance of olaquindox-related residues in foods of animal origin.

## Introduction

Olaquindox (OLQ) has been used as antimicrobial growth promotants (AGPs) for decades to improve feed efficiency and control pig dysentery and bacterial enteritis in young pig. Metabolism studies in rats, chickens, and pigs illustrated that OLQ rapidly converted into monooxy and deoxy metabolites *in vivo*, which could be further biotransformed *via* hydrolysis to 3-methyl-quinoxaline-2-carboxylic acid (MQCA) (1, 2). MQCA was designated as the marker residue for OLQ based on the studies submitted by the sponsor and consideration of the metabolism of the drug (3). Since 1998, OLQ and another quinoxaline antibacterial dug [carbadox (CBX)] have been withdrawn from the market in European Union due to health concerns over possible carcinogenic and mutagenic effects of the drugs and their desoxy metabolites (4). In 2018, due to potential risks to the quality of animal products and public health safety, OLQ was banned for food animals in China. According to the need of regulation, European Reference Laboratory (Fougeres-France) proposed for DCBX, QCA, and MQCA in meat a recommended concentration of 10 μg kg^−1^ as a minimum requirement for analytical methods in 2007 (5).

Up to now, a number of methods reported for monitoring the residues of OLQ have focused on MQCA, including high-performance liquid chromatography with ultraviolet detection (HPLC-UV) (6, 7), GC-ECD (or GC-MS) (8, 9), and LC-MS/MS (10–13). However, these methods always concentrated on the parent drug and MQCA in the absence of residue depletion studies, and none of them described the simultaneous determination of OLQ and its major metabolites in a single run.

**Figure 1:**
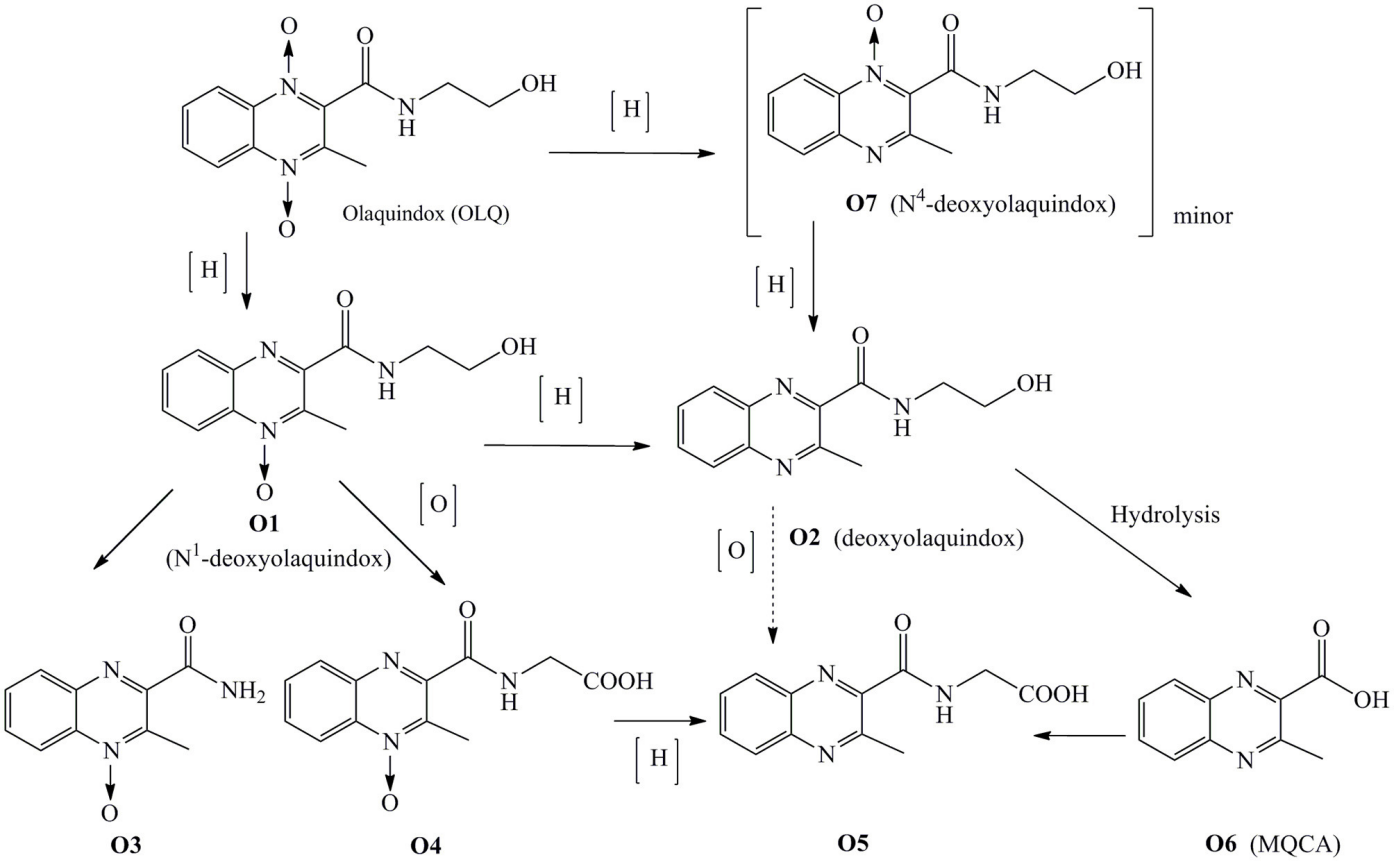
The proposed metabolism pathway of olaquindox in pigs and broilers.

In our laboratory, work on the disposition of ^3^H-olaquindox in pigs, broilers, rats, and carp has been recently carried out by using LC/MS-IT-TOF-*v*.ARC (14). The metabolic pathway of OLQ in pigs and broilers is summarized in Figure 1. The results revealed that the concentration of deoxyolaquindox was higher than those of other metabolites in liver and kidney tissues of pigs and broilers after being fed with a diet containing ^3^H-olaquindox 50 mg kg^−1^ for 14 consecutive days, while, MQCA, the previously designated marker residue, could be detected up to 3 days in the liver and kidney tissues of pig. Therefore, it is reasonable to doubt that whether MQCA is a suitable marker for monitoring carcinogenic metabolites of OLQ. According to the VICH GL 46 guideline, all the major metabolites including that comprising ≥100 μg kg^−1^ or ≥10% of the total residue in a tissue sample should be examined to select the marker residue for monitoring the total residue in the target animal (15).

On the basis of the above observations and guidelines and as a continuation of our research program on the residue depletion of OLQ (14), more residue depletion research should be performed to address the issues related to the target tissue and marker residue of OLQ. In the present study, a sensitive and accurate HPLC-UV method was established for simultaneous determination of OLQ and its six main metabolites (named O1, O2, O3, O4, O5, and O6) in the liver, kidney, muscle, and fat tissues of pigs and broilers. Moreover, residue depletion studies of OLQ in pigs and broilers were investigated to characterize the kinetics of OLQ and its main metabolites in edible tissues, which could provide basic data for the food safety evaluation related to OLQ. To our knowledge, this is the first time that a full residue depletion study is performed for the major metabolites of OLQ in pigs and broilers.

## Materials and Methods

### Chemicals and Reagents

The analytical standards of OLQ, O1, O2, O3, O4, O5, and O6 (>97% purity) were provided by the Institute of Veterinary Pharmaceuticals (Huazhong Agricultural University, Wuhan, People's Republic of China). Individual stock standard solutions (1,000 μg ml^−1^) of all analytes were prepared by dissolving each standard in methanol (MeOH). The mixed standard solution (20 μg ml^−1^) was prepared by combining 2.0 ml of each stock standard and diluting with MeOH to obtain a final volume of 100 ml. The stock solutions were stored in amber vials at −20°C and stabilized for 2 months. The mixed standard solution was stored in an amber vial at 4°C and stabilized for 1 month. Distilled water was further purified by passing through a Milli-Q Plus apparatus (Millipore, Bedford, MA, USA). HPLC-grade MeOH and acetonitrile (MeCN) were purchased from Tedia (Fairfield, OH, USA). Other chemicals, including formic acid and metaphosphoric acid, were of analytical reagent grade.

### Animals and Sampling

The use of animals and all experimental protocols in this study were in accordance with the guidelines of the Committee on the Care and Use of Laboratory Animals of China. Thirty healthy castrated crossbred (Large White × Landrace) pigs (60-day old, 18–20 kg) were purchased from the Breeding Pig Testing Center (Wuhan, China) and housed in six 8 m × 10 m pig pens. Thirty-five 14-day-old Cobb 500 broilers were purchased from Charoen Pokph and Group (Wuhan, China) and kept in stainless steel cages. The animal houses were maintained at 25 ± 2°C room temperature with 45–65% relative humidity. The animals were allowed to acclimate for 7 days before our experiments were conducted. A standard ration based on corn and soybean was fed twice a day. The components in the standard ration for pigs included corn (63%), soybean (27%), bran (6%), and premix (4%), and for broilers, the components included corn (61.4%), soybean (30%), soybean oil (0.6%), carp powder (3%), and premix (5%). Tap water was available *ad libitum*. The animals were randomly divided into a control group (*n* = 5 for both pigs and broilers) and a test group (*n* = 25 and 30 for pigs and broilers, respectively). The control groups were fed with standard ration without OLQ. The treatment groups were provided with medicated feed contained a standard ration premixed with OLQ at a level of 50 mg kg^−1^ diet for 14 consecutive days. At different time points (6 h and 1, 3, 7, and 14 days for pigs and 6 h and 1, 3, 5, and 7 days for broilers), one control animal and five medicated pigs and six medicated broilers were anesthetized with propofol and killed after the last dosing. Samples of liver, kidney, muscle, and fat (skin) were collected. All the collected samples were cut with scissors into small pieces, homogenized and immediately transferred into labeled plastic bags for storage at −20°C pending further analysis.

### Sample Preparation

Each tissue sample (5.0 ± 0.1 g) was thawed and weighed out into a 50-ml polypropylene centrifuge tube. Ten milliliters of 5% (*w*/*v*) metaphosphoric acid in 20% (*v*/*v*) methanol was added to the sample and mixed for at least 1 min by a vortex mixer. After centrifugation at 4,500 × g for 10 min under 5°C, the supernatant was removed into another tube, and the tissue in the tube was extracted again following the above procedure. The supernatants were combined into a 50-ml polypropylene centrifuge tube and defatted with 3 ml hexane. The sample extraction was ready for the cleanup procedure. The HLB cartridge (60 mg, 3 ml) (Waters Corp., Milford, MA, USA) was preconditioned with 3 ml of MeOH followed by 3 ml of water. The extract was added to the HLB cartridge and allowed to flow using gravity. The column was washed with 3 ml of 0.5% formic acid in 5% MeOH (*v*/*v*) and dried by purging air for at least 10 min. Then the analyte was eluted with 6 ml of 90% MeOH (*v*/*v*) at a flow rate of 1.0 ml min^−1^ into a 10-ml tube. The collected elute was evaporated to dryness under a stream of nitrogen at 45°C (N-EVAP™ 112 NITROGEN EVAPORATOR). The residue was reconstituted in 1 ml of 20% MeOH and filtered through a 0.22-μm nylon Millipore chromatographic filter. A 40-μl aliquot was used for HPLC analysis.

### Chromatographic Conditions

The liquid chromatographic separation was performed in a Waters 2695 HPLC system coupled with a Waters 2487 UV detector. Chromatographic separation was achieved on a ZORBAX SB-C18 column (250 mm × 4.6 mm i.d., 5 μm; Agilent Technology, USA) coupled with a C18 guard column at a flow rate of 1.0 ml min^−1^ at 30°C in a column oven. Gradient elution was used for the separation. Initially, the gradient was held for 5 min at 85% mobile phase A (0.6% formic acid in water) and 15% mobile phase B (acetonitrile). The gradient condition was stepped to 10% mobile phase A and 90% mobile phase B in 20 min. The latter condition was maintained for 3 min. The UV detector was set at a wavelength of 320 nm for all of the compounds, and the injection volume was 40 μl.

### Method Validation

The developed method was fully validated according to the EU Commission Decision 2002/657/EC (16). Essential parameters in validating an analytical procedure, such as specificity, linearity, sensitivity, accuracy, precision, and analyte stability, were evaluated to determine the robustness of the developed method.

#### Specificity

The specificity was measured by analyzing 20 blank pigs' or broilers' liver, kidney, muscle, and fat samples using the abovementioned method to evaluate possible endogenous interferences. The results were evaluated based on the presence of interfering substances around the analyte retention time.

#### Calibration Curve and Linearity

The calibration curves were built by spiking blank sample extracts with selected seven concentration levels (20–1,000 μg L^−1^ for O1–O6, 30–500 μg L^−1^ for OLQ). The analyses were performed in triplicate. The standard curve regression equation and correlation coefficient were estimated *via* linear regression using the resultant drug chromatographic peak area (X) and the corresponding drug concentration (Y). The calibration curves constructed on five separate days were analyzed to evaluate the linearity of each curve. Slope, intercept, and correlation coefficient were calculated for each standard curve. Unknown concentrations were calculated from the equation of the calibration curve.

#### Limits of Detection and Limits of Quantification

The limit of detection (LOD) was defined as the lowest concentration of that residue in the sample which can be detected. It is calculated as the mean value of the matrix blank readings plus three standard deviations of the blank (signal-to-noise ratio, S/N = 3:1). The limit of quantification (LOQ) was the lowest fortified sample for which precision and accuracy were determined and found acceptable.

#### Accuracy and Precision

The precision of the method (RSD, %) based on intra-day repeatability was assessed by replicate measurements (*n* = 6) from three spiked tissue samples at three different levels (1, 2, and 4 times of the LOQ). The between-day precision of the method was established using spiked samples at the same concentrations. Five replicates determination of each concentration was conducted over a period of five consecutive days. Accuracy was verified by measuring the recoveries from the spiked blank samples at three concentration levels (1, 2, and 4 times of the LOQ) and five replicates at each fortification level.

#### Stability

The stability of the analytes was assessed under various conditions by using standard solutions, the post-preparation samples and the spiked tissue samples (liver, kidney, muscle and fat of pigs and broilers). The stability of standard solutions was evaluated for 1, 2, and 4 weeks at −20°C. The short-term stability for the postpreparation samples was evaluated after 1, 6, and 24 h storage at 4°C in the dark, and long-term stability was assessed after 1, 3, and 7 days storage at −20 °C in the dark. For the spiked tissue samples, the stability of the analytes was evaluated for 2 and 4 weeks at 4°C and for 1 and 3 months in the deep freezer at −20°C in the dark. Also, the freeze-thaw stability was evaluated after four successive freeze-thaw cycles (−20 to 20°C).

### Statistics

Chemical structure was built by ChemDraw 19.0. Data analysis was performed using GraphPad Prime 7.0. Tissue depletion analysis was using the Microsoft Excel.

## Result and Discussion

### Sample Pretreatment

The sample extraction and cleanup procedure are very important for residue analysis in edible tissues. Though the residue depletion studies of radiolabeled OLQ in pigs showed that no bound residues appeared to be present in tissues, most applied procedures for extraction of the marker residue of OLQ, MQCA from tissues based on acid hydrolysis, alkaline hydrolysis or enzymatic digestion (10–13). Acidic hydrolysis and alkaline hydrolysis procedures require a great caution because of narrow range of pH values, thus a relatively low absolute recovery is anticipated and hence the method precision may also be affected. It was reported that pH had a great impact on the recovery. MQCA and QCA recoveries were low at pH >3 when liquid-liquid extraction (LLE) was used (9). Additionally, because quinoxaline-*N*-oxides are liable to degradation either in alkaline or acidic environment, a mild sample pretreatment procedure is employed to avoid the transformation of analytes. In this study, the sample preparation was according to the previously method (7), 5% (*w*/*v*) metaphosphoric acid in 20% (*v*/*v*) methanol was used as extraction solvent which could liberate the carboxylic compounds (O4, O5, and O6), as well as remove the protein. The results demonstrated that higher recoveries of all the analytes were obtained without noticeable degradation.

Solid phase extraction (SPE) was essential for clean-up of the tissue samples in order to effectively purify the tissue extracts for multiple components analysis. OLQ was biotransformed into a series of deoxylation, oxidation and hydrolysis metabolites, which were differ in polarity and chemical properties. In the present study, when Oasis HLB was chosen for the SPE procedure, 5% (*w*/*v*) metaphosphoric acid was used for pH adjustment to 2–3, and washing solvent was found to effectively remove the interferences and provide sample clean-up without eluting the analytes. The same as in the HPLC mobile phase, addition of formic acid could suppress ionization of the carboxylic compounds, which could prolong the retention time on the C18 column and improve the peak shapes of the analytes.

### Method Validation

**Figure 2:**
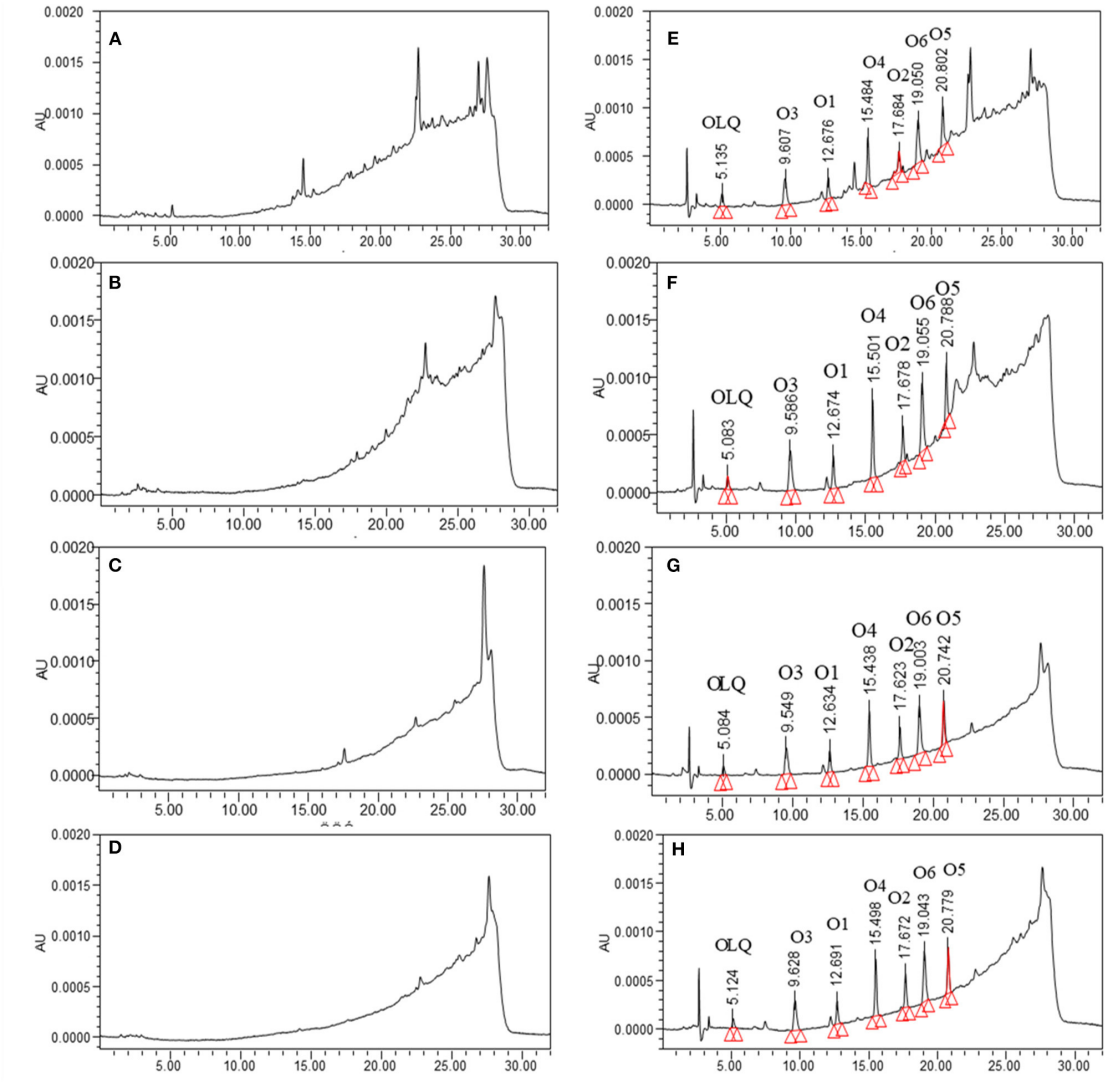
The chromatograms of blank samples from pigs: **(A)** liver, **(B)** kidney, **(C)** muscle, **(D)** fat, and corresponding blank samples spiked with olaquindox and its main metabolites (40 μg kg^−1^): **(E)** liver, **(F)** kidney, **(G)** muscle, **(H)** fat.

The specificity of the method was assessed by preparation and analysis of 20 blank pigs' or broilers' liver, kidney, muscle, and fat samples. The results did not show any significant endogenous interference near the retention time of the products for all analytes (Figure 2). The calibration curves were linear with coefficients (*r*^2^) >0.99 for all analytes. In this method, the LOD of 5 μg kg^−1^ for O1 and O2, and 10 μg kg^−1^ for OLQ, O3, O4, O5, and O6 was established in all pigs' and broilers' tissues. The LOQ of O1 and O2 was 10 μg kg^−1^ in liver, kidney, muscle, and fat tissues of pigs and broilers, and that of O3, O4, O5, and O6 was 20 μg kg^−1^, while that of OLQ was 30 μg kg^−1^. Intraday RSD and interday RSD were 2.6–8.65% and 3.5–12.56%, respectively. The absolute recovery was calculated by comparing the observed concentration with the spiked concentration. The study indicated that the mean recoveries ranged from 63.5 to 91.5% for all the analytes. Regarding the storage stability of the analytes, the standard solutions of these analytes in MeOH were stable for at least 14 days when stored at −20°C in the dark. The postpreparation samples were stable for 24 h at 4°C and for 7 days of storage at −20°C in the dark. For short-term stability test, the analytes in spiked tissue samples (liver, kidney, muscle, and fat) were stable for 2 weeks at 4°C in the dark. For long-term stability, the analytes were found stable for 3 months of storage at −20°C in the dark, and they were stable after four freeze-thaw cycles from −20°C to room temperature.

### Characteristics of OLQ Residues Depletion in Pigs and Broilers

OLQ has been proved to be effective in control pig dysentery and bacterial enteritis, and it is restricted to use only in young pigs. However, extra label use of drugs often occurs in China, thus the hazard residues related to OLQ in broilers should also be concerned to ensure the food safety. In this study, a comparative study on the tissue depletion of OLQ and its six main metabolites in pigs and broilers were performed.

The mean residue concentrations of OLQ and its main metabolites in the edible tissues (liver, kidney, muscle, and fat) of pigs and broilers after being fed with OLQ for 14 consecutive days were presented in Table 1. The results showed that OLQ eliminated rapidly from the tissues of pigs and broilers postremoval of medicated feed. OLQ and its main metabolites residues were all found in liver and kidney tissues of pigs and broilers at 6 h postremoval of medicated feed. After that, OLQ and its mono deoxy metabolite (O1) eliminated quickly and could not be detected after 1 day postmedication. A maximum concentration of 934.0 μg kg^−1^ for the deoxy metabolite (O2) was found in the kidney of pigs at 6 h, which declined to 111.0 μg kg^−1^ at the withdrawal time of 7 days. In pig liver tissues, the concentration of O2 decreased from 317.1 μg kg^−1^ at 6 h to 86.5 μg kg^−1^ at 3 days postremoval of medicated feed and was below the quantification limit after 7 days. The designated marker residue (O6) also could be detected at the withdrawal time of 7 days in pig kidneys with a concentration of 45.6 μg kg^−1^, while in pig liver tissues, it lasted only for 3 days with a concentration of 35.8 μg kg^−1^. OLQ and the residues of its main metabolites were relatively less in pig muscle and fat tissues with concentrations ranging from 21.3 to 96.1 μg kg^−1^. At 3 days postmedication, O2 was the only residue that could be detected in the muscle and fat tissues of pigs. Two oxidation metabolites (O4 and O5) were found at relatively high concentrations in pig kidney tissues at 6 h postmedication (737.6 and 253.4 μg kg^−1^, respectively), but they eliminated rapidly and could not be detected at 7 days postmedication.

**Table 1:** Concentrations of olaquindox and its metabolites in edible tissues of pigs (*n* = 5) and broilers (*n* = 6) at different days after being fed with olaquindox for 14 consecutive days.

**Tissue**	**Time (days)**	**Compounds (μg kg**^****−1****^**)**						
		**OLQ**	**O1**	**O2**	**O3**	**O4**	**O5**	**O6**
**Pigs**								
Liver	0.25	112.4 ± 38.1	86.2 ± 25.3	317.1 ± 75.3	60.5 ± 10.4	137.6 ± 8.5	106.4 ± 11.0	126.5 ± 15.3
	1	ND	ND	182.4 ± 46.2	ND	44.2 ± 10.8	80.6 ± 11.0	81.6 ± 12.4
	3	ND	ND	86.5 ± 22.8	ND	ND	ND	43.8 ± 10.5
	7	ND	ND	23.6	ND	ND	ND	ND
	14	ND	ND	ND	ND	ND	ND	ND
Kidney	0.25	142.3 ± 30.6	121.1 ± 39.5	934.0 ± 125.9	67.4 ± 39.7	737.6 ± 99.2	253.4 ± 41.1	402.8 ± 71.3
	1	ND	85.5 ± 17.3	625.8 ± 112.0	ND	280.9 ± 49.9	129.4 ± 22.8	230.0 ± 33.1
	3	ND	ND	293.6 ± 55.0	ND	72.5 ± 15.0	48.5 ± 12.8	101.0 ± 16.3
	7	ND	ND	111.0 ± 24.6	ND	ND	ND	45.6 ± 11.5
	14	ND	ND	ND	ND	ND	ND	ND
Muscle	0.25	68.4 ± 15.5	39.6 ± 10.4	96.1 ± 21.2	31.5 ± 8.4	21.3 ± 0.8	36.2 ± 6.4	67.6 ± 11.3
	1	ND	ND	62.8 ± 10.5	ND	ND	ND	42.2 ± 8.8
	3	ND	ND	29.2 ± 7.5	ND	ND	ND	ND
	7	ND	ND	ND	ND	ND	ND	ND
Fat	0.25	57.5 ± 18.1	36.6 ± 6.8	63.7 ± 16.2	ND	ND	32.3 ± 5.4	52.6 ± 10.3
	1	ND	ND	42.9 ± 11.2	ND	ND	ND	ND
	3	ND	ND	ND	ND	ND	ND	ND
**Broilers**								
Liver	0.25	298.9 ± 53.6	178.1 ± 32.3	133.3 ± 28.8	56.5 ± 13.8	51.1 ± 12.6	62.7 ± 12.9	80.6 ± 19.8
	1	136.2 ± 35.0	52.9 ± 10.9	81.1 ± 20.1	ND	29.2 ± 7.8	44.1 ± 7.9	67.3 ± 12.2
	3	ND	ND	58.6 ± 11.9	ND	ND	ND	27.3 ± 7.5
	5	ND	ND	43.6 ± 9.7	ND	ND	ND	ND
	7	ND	ND	ND	ND	ND	ND	ND
Kidney	0.25	253.4 ± 43.6	170.1 ± 41.6	105.0 ± 21.9	73.5 ± 21.3	48.7 ± 14.2	61.6 ± 21.9	106.6 ± 31.3
	1	101.6 ± 16.9	49.7 ± 8.2	71.9 ± 15.6	ND	27.9 ± 6.2	40.6 ± 12.5	72.5 ± 23.5
	3	ND	ND	57.7 ± 18.7	ND	ND	24.2 ± 8.5	38.5 ± 11.0
	5	ND	ND	40.5 ± 12.8	ND	ND	ND	ND
	7	ND	ND	ND	ND	ND	ND	ND
Muscle	0.25	75.7 ± 26.3	48.6 ± 20.0	68.1 ± 30.9	ND	ND	ND	28.4 ± 12.3
	1	ND	23.3 ± 8.6	28.4 ± 6.5	ND	ND	ND	ND
	3	ND	ND	ND	ND	ND	ND	ND
Fat	0.25	51.8 ± 9.2	38.2 ± 11.3	37.6 ± 12.2	ND	ND	ND	23.1 ± 10.3
	1	ND	ND	ND	ND	ND	ND	ND
	3	ND	ND	ND	ND	ND	ND	ND

*ND, less than the LOQ or not detected*.

In broilers, all analytes could be detected in liver and kidney. However, there were obvious differences between broilers and pigs. The parent drug was the primary residue presented in liver and kidney samples at 6 h postmedication, in which the concentration was 298.9 and 253.4 μg kg^−1^, respectively. The residue with the next highest concentration was O1 in liver and kidney tissues, which was about 170 μg kg^−1^ at the same time point. However, both of them eliminated quickly from the tissues and could not be detected at the withdrawal time of 3 days. As in pigs, the deoxy metabolite (O2) residues in broiler tissues were still more persistent than other metabolites. After 5 days, O2 residue remained 43.6 and 40.5 μg kg^−1^ in liver and kidney, respectively, while the others were not found in any other tissue. O6 residues in broiler tissues were less than those in pigs. A maximum concentration of 106.6 μg kg^−1^ was found in broiler kidneys, and it decreased below the limit of quantification after 3 days postmedication.

**Figure 3:**
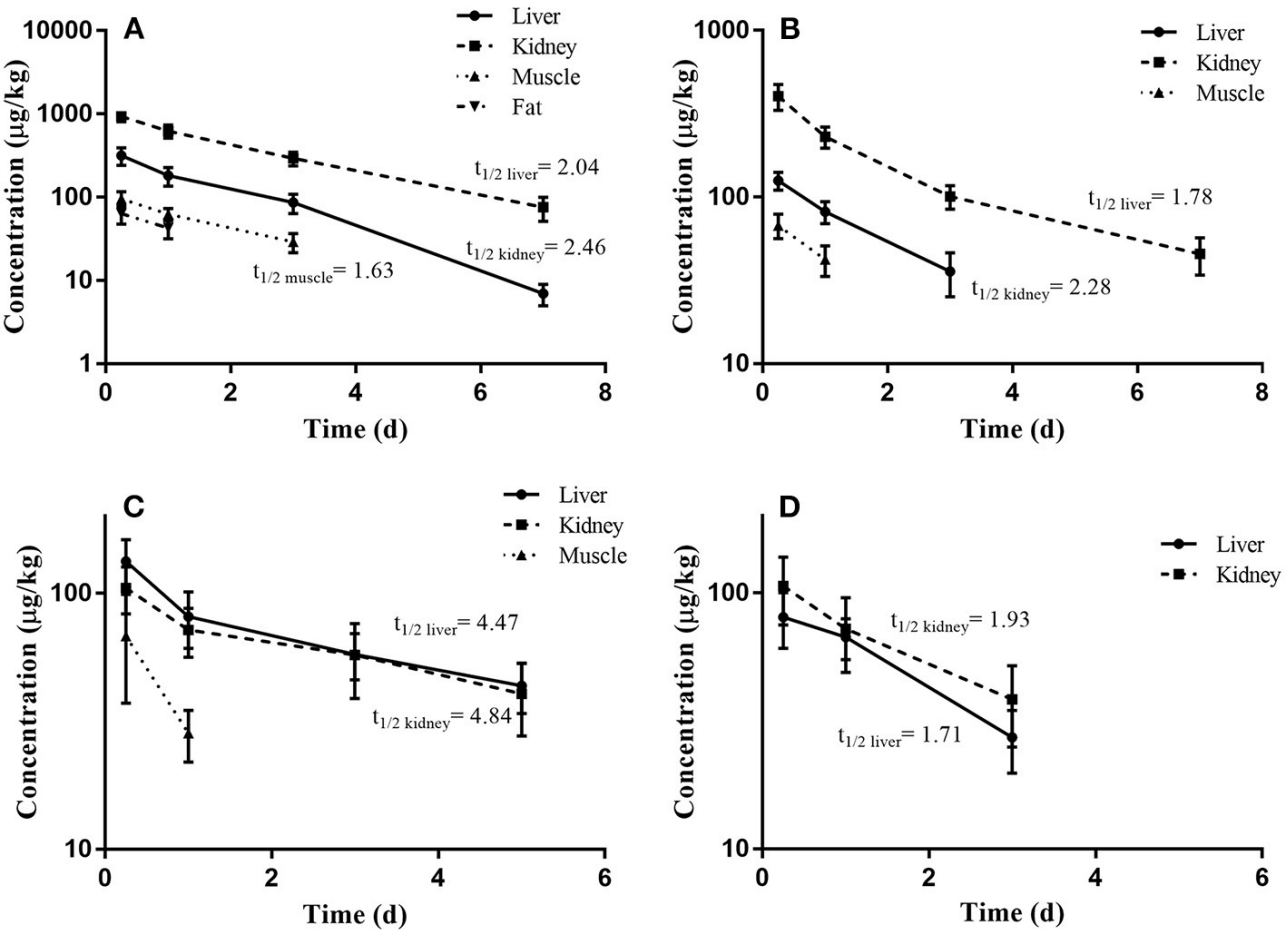
The depletion plots of mean concentrations of O2 and O6 in the edible tissues of pigs and broilers slaughtered at different days following continuous feeding of olaquindox for 14 days (mean ± SD): **(A)** O2, **(B)** O6 in pig tissues; **(C)** O2, **(D)** O6 in broiler tissues.

The depletion plots of mean concentrations of O2 and O6 in the liver, kidney, muscle, and fat tissues of pigs and broilers are illustrated in Figure 3. The elimination half-lives (*t*_1/2k_) of OLQ and its main metabolites were determined in individual tissues assuming a single compartment model and first-order kinetics. The last three time-point data were fit to the first-order rate equation *C*_*t*_ = *C*_0_ e^−*kt*^, where *C*_*t*_ were the concentrations of OLQ and its main metabolites on day *t, C*_0_ was the initial concentration, elimination rate constant (*k*) was the slope of the linear regression equation for the log-transformed residue concentration (ln C) against time, and the half-life of elimination (*t*_1/2k_) was calculated from the equation *t*_1/2k_ = ln 2/*k* for each tissue. The calculated *t*_1/2k_ of O2 and O6 in the kidney tissue of pigs were 2.46 and 2.28 days, respectively, which was longer than that in liver tissues. The results we obtained were similar to the previous study (14); they found that the radioactivities could be detected in the liver and kidney until 14 days, but a higher amount of radioactivities could be detected in the kidney. Therefore, they indicated kidney as the target tissue of OLA residues in pigs. An elimination half-life of 1.63 days for O2 in muscle of pigs was also obtained. The calculated *t*_1/2k_ of O6 in the liver and kidney of broilers (1.71 and 1.93 days, respectively) was similar to that of pigs. However, the elimination half-lives of O2 in the liver and kidney of broilers were 4.47 and 4.84 days, respectively, which were much longer than those of pigs (Table 2). The work by Tan et al. also demonstrates that O2 persisted the longest time in the kidney with a half-life of 3.59 days in broilers, and thus O2 was recommended as the marker residue of OLQ (14). The elimination rates of olaquindox-related residues in pig tissues were found to be in the following order: kidney < liver < fat and muscle. Similar trends were found in broilers, but the difference in elimination rate between liver and kidney was not apparent. The multiple residue depletion data indicated that kidney and liver could be more appropriate target tissues and O2 was the most relevant marker residue for OLQ related residue monitoring. Similar situations have been found in another quinoxaline antibacterial drug, carbadox (CBX). Quinoxaline-2-carboxylic acid (QCA) was designated as the marker residue for CBX (1). However, later studies showed that desoxycarbadox, the suspect carcinogen, persisted in animal tissues when the concentration of QCA had reached the MRL. Accordingly, JECFA concluded that QCA was not a suitable marker residue for CBX in 2003 (17). A later study demonstrated that QCA was not a suitable marker residue for CBX, and the deoxy metabolites, desoxycarbadox, should be monitored for the regulation of CBX in food animal production (12).

**Table 2:** The elimination half-lives of O2 and O6 in the tissues of pigs and broilers after being fed with olaquindox for 14 consecutive days.

**Tissue**	**Elimination half-life (days)**			
	**Pigs**		**Broilers**	
	**O2**	**O6**	**O2**	**O6**
Liver	2.04	1.78	4.47	1.71
Kidney	2.46	2.28	4.84	1.93
Muscle	1.63	–	–	–

The results of this residue depletion study demonstrated that the elimination half-lives of O6 in broiler liver and kidney tissues were similar to those of pigs, but the O6 concentrations were lower than those in pig tissues. O2 concentrations in broiler tissues were also much lower than those in pig tissues, while much longer elimination half-lives of O2 (>4 days) were found in kidney and liver tissues. For this reason, the use of OLQ in broilers is apparently inappropriate, as significant drug residue will occur. Moreover, it has been reported that the main toxicities of OLQ were hepatotoxicity and nephrotoxicity (18, 19). In China, many clinical cases report that poultry are more susceptible than pigs to poisoning after OLQ administration. In this study, it was found that the residue level of the parent drug and its mono deoxy metabolite (O1) in the liver and kidney of broilers was significantly higher than that in pigs, while deoxyolaquindox concentrations were much lower in broilers' tissues than those in pig tissues. The results indicated that the enzymatic metabolism in pigs had greater reducing capacity and could reduce the nitrogen-oxygen group of OLQ more rapidly and effectively than in broilers. Meanwhile, the concentrations of two oxygenated metabolites (O4 and O5) in pig tissues were also found higher than those in broiler tissues. Since the compound with carboxyl group may be highly polar and thus easily excreted from tissue, we deduce that the oxidase in pigs, which can biotransform the hydroxyl group into carboxyl group, is more effective than that in broilers. The metabolic pathways of OLQ, either by deoxylation into deoxy metabolites or by oxidation into carboxyl metabolites, could be seemed as a detoxification process. From this perspective, it should be stressed that the use of OLQ as AGP in broilers is inappropriate.

## Conclusion

The results of this study demonstrated that OLQ was biotransformed into six major metabolites through *in vivo* reduction, hydrolysis, and oxidation processes in pigs and broilers after being fed with OLQ for 14 consecutive days. The depletion studies showed that the deoxyolaquindox occurred at higher concentrations and was more persistent than other residues in edible tissues. MQCA, the previously designated marker residue, was detected at lower levels and eliminated faster than deoxyolaquindox in all detected tissues. Thus, deoxyolaquindox is the most relevant marker residue. Moreover, because the elimination half-lives of deoxyolaquindox residue in broilers liver and kidney tissues are much longer than those in pigs. Thus, the use of OLQ should also be forbidden in broilers, due to the presence of significant drug residues. All these findings can help to establish more reasonable and effective standards for residual markers of OLQ, which is crucial to maintain a powerful effect on drug regulation.

## Data Availability Statement

The original contributions generated for the study are included in the article/supplementary material, further inquiries can be directed to the corresponding author/s.

## Ethics Statement

The animal study was reviewed and approved by Institutional Animal Care and Use Committee protocols at Huazhong Agricultural University.

## Author Contributions

YP conceived the idea. HZ and YP constructed the workflow. HZ and WQ performed the experiments and completed the paper. CD and JH analyzed and discussed the data. YP and ZY revised the paper. SX, ZL, and LH performed and revised the experiments. All authors discussed the results and contributed to the final manuscript.

## Conflict of Interest

The authors declare that the research was conducted in the absence of any commercial or financial relationships that could be construed as a potential conflict of interest. The reviewers (YW and DP) declared a shared affiliation with the authors, to the handling editor at time of review.

## References

[1] FAO/WHO. Joint Expert Committee on Food Additives: Evaluation of Certain Veterinary Drug Residues in Food, Technical Series 799. Geneva (1990). 2124403

[2] Liu ZY Huang LL Zhou XN Chen DM Tao YF Zhang HH. The metabolism of olaquindox in rats, chickens and pigs. Toxicol Lett. (2011) 200:24–33. 10.1016/j.toxlet.2010.10.010 20974235

[3] FAO/WHO. Joint Expert Committee on Food Additives: Evaluation of Certain Veterinary Drug Residues in Food, Technical Series 851. Geneva (1995). 7597817

[4] European Commission. Commission Regulation (EC) No. 2788/98. OJEC. (1998) L347:3.

[5] CRL Guidance. CRLs View on State of the Art Analytical Methods for National Residue Control Plans (2007). Available online at: www.rivm.nl/bibliotheek/digitaaldepot/crlguidance2007.pdf

[6] Nagata T Saeki M. Determination of olaquindox residues in swine tissues by liquid chromatography. J Assoc Off Anal Chem. (1987) 70:706–7. 10.1093/jaoac/70.4.706 3624181

[7] Wu Y Yu H Wang Y Huang L Tao Y Chen D. Development of a high-performance liquid chromatography method for the simultaneous quantification of quinoxaline-2-carboxylic acid methyl-3-quinoxaline-2-carboxylic acid in animal tissues. J Chromatogr A. (2007) 1146:1−7. 10.1016/j.chroma.2006.11.024 17335836

[8] Lynch MJ Bartolucci SR. Confirmatory identification of carbadox-related residues in swine liver by gas-liquid chromatography/mass spectrometry with selected ion monitoring. J Assoc Off Anal Chem. (1982) 65:66–70. 10.1093/jaoac/65.1.66 7056693

[9] Sin D Chung L Lai M Siu S Tang H. Determination of quinoxaline-2-carboxylic acid, the major metabolite of carbadox, in porcine liver by isotope dilution gas chromatography-electron capture negative ionization mass spectrometry. Anal Chim Acta. (2004) 508:147–58. 10.1016/j.aca.2003.11.067

[10] Hutchinson MJ Young PY Hewitt SA Faulkner D Kennedy DG. Development and validation of an improved method for confirmation of the carbadox metabolite, quinoxaline-2-carboxylic acid, in porcine liver using LC-electrospray MS-MS according to revised EU criteria for veterinary drug residue analysis. Analyst. (2002) 127:342–6. 10.1039/b109425b 11996357

[11] Hutchinson MJ Young PB Kennedy DG. Confirmation of carbadox and olaquindox metabolites in porcine liver using liquid chromatography-electrospray, tandem mass spectrometry. J Chromatogr B Analyt Technol Biomed Life Sci. (2005) 816:15–20. 10.1016/j.jchromb.2004.09.024 15664328

[12] Boison JO Lee SC Gedir RG. A determinative and confirmatory method for residues of the metabolites of carbadox and olaquindox in porcine tissues. Anal Chim Acta. (2009) 637:128–34. 10.1016/j.aca.2008.09.016 19286021

[13] Sniegocki T Gbylik-Sikorska M Posyniak A Zmudzki J. Determination of carbadox and olaquindox metabolites in swine muscle by liquid chromatography/mass spectrometry. J Chromatogr B. (2014) 944:25–9. 10.1016/j.jchromb.2013.09.039 24291715

[14] Tan H Pan Y Chen D Tao Y Zhou K Liu Z. Discovery of the marker residue of olaquindox in pigs, broilers, and carp. J Agr Food Chem. (2019) 67:6603–13. 10.1021/acs.jafc.8b06026 31094200

[15] VICH. Studies to Evaluate the Metabolism and Residue Kinetics of Veterinary Drugs in Food-producing Animals: Metabolism Study to Determine the Quantity and Identify the Nature of Residues (MRK), VICH GL 46 Guideline. Rockville (2011).

[16] Commission Decision. Commission decision 2002/657/EC of 12 August 2002 implementing council directive 96/23/EC concerning the performance of analytical methods and interpretation of results. Off J Eur Commun. (2002) L.221:8–23.

[17] FAO/WHO. Joint Expert Committee on Food Additives: Evaluation of Certain Veterinary Drug Residues in Food, Technical Series 918. Geneva (2003).

[18] Huang XJ Zhang HH Wang X Huang LL Zhang LY Yan CX. ROS mediated cytotoxicity of porcine adrenocortical cells induced by QdNOs derivatives *in vitro*. Chem Biol Interact. (2010) 185:227–34. 10.1016/j.cbi.2010.02.030 20188712

[19] Wang X Wan D Ihsan A Liu Q Cheng G Li J. Mechanism of adrenocortical toxicity induced by quinocetone and its bidesoxy-quinocetone metabolite in porcine adrenocortical cells *in vitro*. Food Chem Toxicol. (2015) 84:115–24. 10.1016/j.fct.2015.08.016 26296292

